# Discovery of therapeutic targets of quercetin for endometrial carcinoma patients infected with COVID-19 through network pharmacology

**DOI:** 10.3389/fonc.2023.1151434

**Published:** 2023-03-08

**Authors:** Kehan Li, Hejing Liu, Yibin Lin, Liang Gu, Xinli Xiang, Xueqiong Zhu

**Affiliations:** ^1^ Center of Uterine Cancer Diagnosis & Therapy Research of Zhejiang Province, Department of Obstetrics and Gynecology, The Second Affiliated Hospital of Wenzhou Medical University, Wenzhou, Zhejiang, China; ^2^ Department of Obstetrics and Gynecology, Taizhou Women and Children’s Hospital of Wenzhou Medical University, Taizhou, Zhejiang, China

**Keywords:** COVID-19, uterine corpus endometrial carcinoma (UCEC), quercetin, ubiquitination, network pharmacology (NP)

## Abstract

**Purpose:**

Aimed to identify the anti-uterine corpus endometrial carcinoma (UCEC) function and characterize the mechanism of quercetin in the treatment of patients infected with COVID-19 *via* integrated *in silico* analysis.

**Methods:**

The Cancer Genome Atlas and Genotype Tissue Expression databases were applied to obtain differentially expressed genes of UCEC and non-tumor tissue. Several *in silico* methods such as network pharmacology, functional enrichment analysis, Cox regression analyses, somatic mutation analysis, immune infiltration and molecular docking were used to investigate and analysis the biological targets, functions and mechanisms of anti-UCEC/COVID-19 of quercetin. Multiple methods such as CCK8 assay, Transwell assay and western blotting were performed to test proliferation, migration, and protein level of UCEC (HEC-1 and Ishikawa) cells.

**Results:**

Functional analysis disclosed that quercetin against UCEC/COVID-19 mainly by ‘biological regulation’, ‘response to stimulus’, and ‘regulation of cellular process’. Then, regression analyses indicated that 9 prognostic genes (including *ANPEP*, *OAS1*, *SCGB1A1*, *HLA*‐*A*, *NPPB*, *FGB*, *CCL2*, *TLR4*, and *SERPINE1*) might play important roles in quercetin for treating UCEC/COVID-19. Molecular docking analysis revealed that the protein products of 9 prognostic genes were the important anti-UCEC/COVID-19 biological targets of quercetin. Meanwhile, the proliferation and migration of UCEC cells were inhibited by quercetin. Moreover, after treatment with quercetin, the protein level of ubiquitination-related gene *ISG15* was decreased in UCEC cells *in vitro*.

**Conclusions:**

Taken together, this study provides new treatment option for UCEC patients infected with COVID-19. Quercetin may work by reducing the expression of *ISG15* and participating in ubiquitination-related pathways.

## 1 Introduction

Severe acute respiratory syndrome coronavirus 2 (SARS-CoV-2) is a rapidly emerging global virus which leads to coronavirus disease in 2019 (COVID-19). The search for COVID-19-fighting drugs has been a long and arduous one for researchers. Despite promising results from *in vitro* trials, the antimalarial drug hydroxychloroquine couldn’t prevent symptomatic infection caused by SARS-CoV-2 (1). Similarly, a recent report found amubarvimab plus romlusevimab showed no effectiveness in improving clinical outcomes for hospitalized COVID-19 patients (2). Although nonsteroidal anti-inflammatory drugs could provide partial relief, they may increase the risk of bacterial infection (3). Therefore, it is still imperative to continue the search for drugs treating COVID-19.

Uterine corpus endometrial carcinoma (UCEC) is the most common gynecologic cancer in the high-income countries. In 2021, the numbers of new cases and deaths in the United States were approximately at 66,570 and 12,940, respectively (4). A recent study found that severe events were more likely to occur in cancer patients with COVID-19, compared with those who without cancer in China (5). During the hospitalization of COVID-19 patients, the 30-day mortality was 30% for cancer patients (6) and 21% for non-cancer patients (7). Cancer patients were more susceptible to COVID-19 (8). Theoretically, patients with UCEC are at high risk for COVID-19 infection. The prevalence of COVID-19 in UCEC patients will have a great possibility to diminish patient treatment outcomes and affect prognosis. Consequently, it is necessary to screen and develop medicine for anti-UCEC/COVID-19.

Quercetin is a natural compound finding in flowers, leaves, and seeds of plants. Lin et al. (9) showed that quercetin had strong virus inhibition and anti-inflammatory activity. Quercetin may intervene in events associated with cancer development and progression by promoting apoptosis, inhibiting cell proliferation and cancer-related inflammation (10). Our previous study found quercetin could enhance the effectiveness of cisplatin in cervical cancer (11). Additionally, it was found that a reduction in UCEC risk with quercetin intake (12). Unfortunately, the molecular mechanisms of quercetin on UCEC remains poorly understood.

Dysregulation of ubiquitination leads to uncontrolled cell proliferation and aberrant changes of suppressor or oncogene expression (13). *ISG15*, the first discovered ubiquitin-like protein, was identified to contribute to cancer progression through regulating the ubiquitination (14). Zhao et al. (15) found *ISG15*, which related to poor prognosis, was observed overexpression in UCEC. Interestingly, quercetin treatment was reported to modulate *ISG15* expression (16).

**Figure 1:**
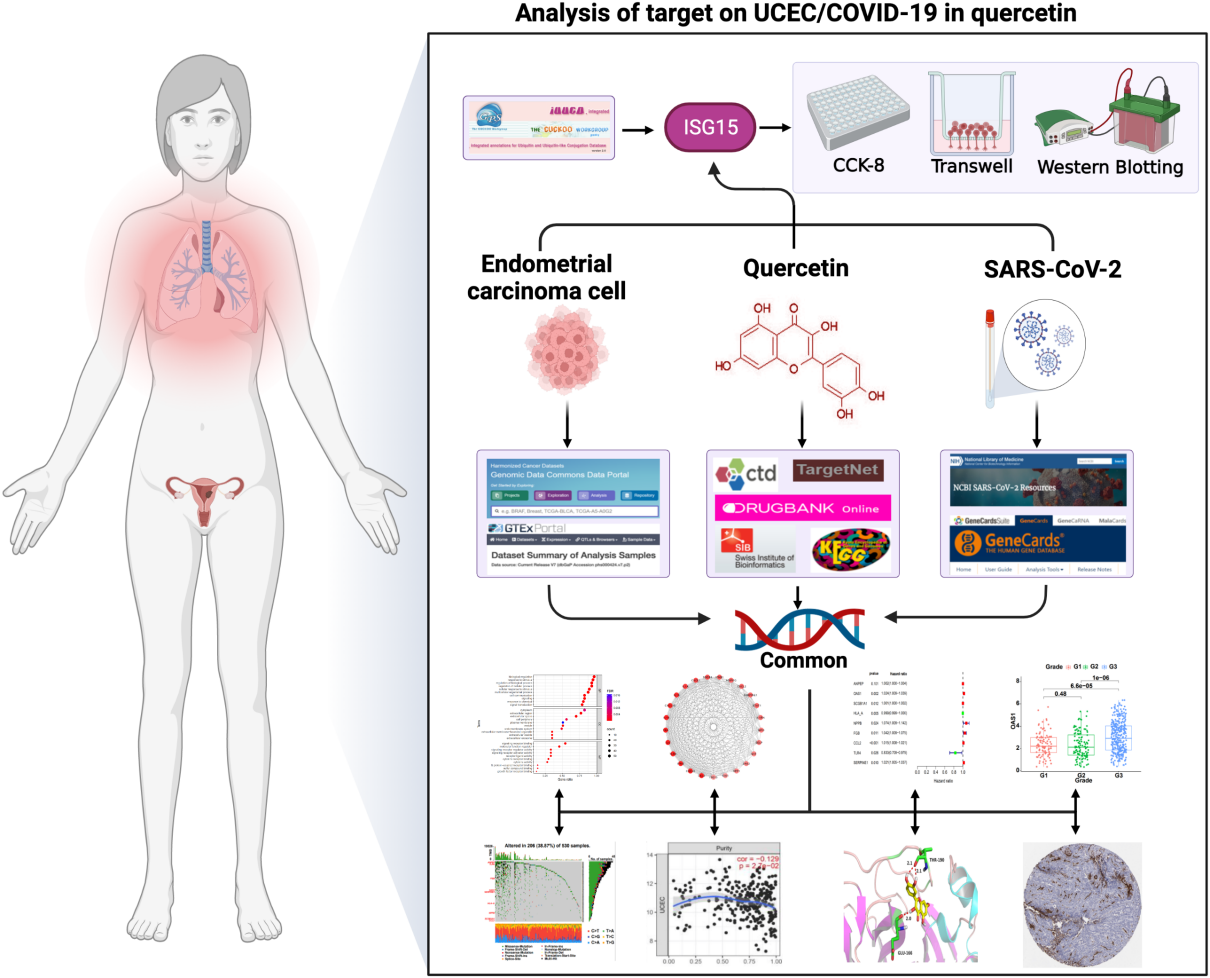
The graphical abstract of this article. The figure represents the antiviral action and mechanism of quercetin against UCEC/COVID-19 using the network pharmacology and computational bioinformatics analysis approach.

Network pharmacology analyzes the link between drugs, targets, and diseases in the same network. For example, Zhang et al. (17) discovered 76 active ingredients of Xihuang pill through network analysis and identified genes and pathways associated with the triple-negative Breast Cancer. Liu et al. (18) screened 8 active ingredients in honeysuckle associated with acute lung injury by constructing a network of ingredients and targets. Furthermore, our previous study found the common targets between drugs for the treatment of cervical cancer through synergistic action (11). These existing studies suggest that it will be possible to explore quercetin for treating UCEC and COVID-19 with the use of network pharmacology. By network pharmacology analysis, this work investigated quercetin’s targets for treating UCEC/COVID-19 and elucidated its mechanisms. Our study provides a theoretical basis for the treatment of quercetin in UCEC/COVID-19 patients. **Figure 1** illustrates the process.

## 2 Materials and methods

### 2.1 Collecting data

The RNA expression profiles, somatic mutation data, and clinical information were downloaded from The Cancer Genome Atlas (TCGA-UCEC cohort). The dataset included 552 UCEC samples and 35 paracancer tissues. Data with missing clinical information were excluded from the corresponding analyses. As a supplement, RNA expression profiles of 142 normal uterus samples from Genotype Tissue Expression (GTEx) were also downloaded.

### 2.2 Identification of UCEC DEGs and COVID-19 related genes

After removing batch effects, differentially expressed genes (DEGs) of UCEC samples and non-tumor tissues were screened by using ‘DEseq2’ package in R language. Additionally, NCBI SARS-CoV-2 Data and Genecards were searched to find COVID-19 related genes (CRGs). Then, the overlapping genes were obtained by comparing the UCEC-DEGs with CRGs.

### 2.3 Acquisition of quercetin targets in UCEC/COVID-19

Five online databases, including Comparative Toxicogenomics Database (CTD), SwissTargetPrediction, DrugBank, Kyoto Encyclopedia of Genes and Genomes (KEGG), and TargetNet were used to identify quercetin targets. After deduplication, the common genes among targets of quercetin, UCEC-DEGs, and CRGs were identified as the main targets of quercetin for anti-UCEC/COVID-19.

### 2.4 Construction of protein-protein interaction (PPI) network

In order to construct the interaction network, PPI prediction was performed by the STRING. Then, the PPI network was evaluated and visualized using Cytoscape (v3.6.1) (19) software. As shown in **Supplementary Table 1**, the MCODE algorithm in Cytoscape was further used to identify the hub genes.

### 2.5 Functional enrichment analyses

In order to investigate the biological function of therapeutic targets of quercetin on UCEC/COVID-19, Gene Ontology (GO) and KEGG pathway analyses were performed. GO enrichment analysis consists of three parts: molecular functional analysis (MF), biological process analysis (BP), and cellular component analysis (CC). KEGG analysis helps us to discover the potential disease-specific pathways. In this study, the genes were entered into the g:Profiler online site to obtain the results of GO and KEGG analyses.

### 2.6 Clinical analysis of core targets for quercetin and UCEC/COVID-19

Univariate Cox analysis was conducted to evaluate the relationship between each quercetin target in UCEC/COVID-19 and survival status of UCEC patients. Subsequently, the obtained genes were analyzed by multivariate Cox analysis. For each patient, there is a formula for calculating their risk score. $risk\;score=\sum_{i}^{9}\left(Coefi*Genei\right)$ (Coef: coefficients, Gene: expression level of gene). The UCEC patients were classified into two risk groups based on the prognostic risk score at the median. Moreover, the overall survival (OS) time of two subgroups were compared utilizing the R packages ‘survival’. The ROC analysis was conducted to test the sensitivity and specificity of prognostic signatures using the ‘timeROC’ package. Additionally, we analyzed the relationship between risk scores and different clinical characteristics, such as age (≤ 65 and > 65), pathological grade, and clinical stage.

### 2.7 Somatic variation, immune infiltration, and protein level analysis of risk signatures

First, somatic mutations of the risk signatures in UCEC were analyzed and visualized by ‘maftools’ package. Then, TIMER database was performed to evaluate the relationship between infiltration of immune cell and risk signatures through xcell algorithm. In order to explore the different protein levels in UCEC tissue and normal endometrial tissue, immunohistochemistry staining images were acquired from Human Protein Atlas (HPA).

### 2.8 Molecular docking analysis

As a critical technique of network pharmacology, molecular docking could predict the binding activity of proteins and molecule compounds (20). Through Protein Data Bank (PDB) database, crystal structure of SARS-CoV-2 main protease (PDB code: 7BQY) was obtained. The Grid Box parameter in AutoDockTools was set to include all receptor areas. AutoDock Vina was used to proceed molecular docking. Docking result between quercetin and 7BQY was visualized by PyMOL (21) software.

### 2.9 Identification of ubiquitination related genes (URGs) in quercetin and UCEC/COVID-19

URGs were obtained from IUUCD database. Then, the intersecting genes were obtained by comparing the URGs and the PPI hub genes.

### 2.10 Cell culture

UCEC cells (HEC-1 and Ishikawa) were obtained from the Cell Bank of the Chinese Academy of Sciences (Shanghai, China). HEC-1 cells were cultured in McCoy’s 5A medium (Gibco, USA) and Ishikawa cells were cultured in dulbecco’s modified Eagle’s medium (DMEM, Gibco, USA), both supplemented with 10% fetal bovine serum (FBS; Gibco, USA) at 37°C with 5% CO2.

### 2.11 Cell viability measurement

CCK-8 kit (meilunbio, China) was used to assess the viability of UCEC cells. Quercetin (Sigma, USA) was dissolved in dimethylsufoxide (DMSO) previously stored at -20 °C. The HEC-1 and Ishikawa cells (5 × 10^3^ cells/well) were dispensed in 96-well flat-bottomed plates and treated with different quercetin concentrations (0, 20, 40 and 80 μM for HEC-1 cells, 0, 25, 50 and 100 μM for Ishikawa cells). The Microplate Reader (Bio Tek Instruments, USA) was applied to determine the absorbance at 450 nm.

### 2.12 Transwell migration assay

For migration assay, the transwell chambers with polycarbonate membranes (8 µm pore, Corning, USA) were used. After treated with quercetin for 48h, the HEC-1 cells (8 × 10^4^ cells/well) and Ishikawa cells (4 × 10^4^ cells/well) were planted in the upper compartment in the 24-well plate with 100 µL FBS-free medium, while the lower compartment was using DMEM with 10% FBS. The membranes were fixed with 4% paraformaldehyde after incubating 24 h, and then stained with 0.1% crystal violet dye for 10 min. By using the inverted routine microscope (Leica, Japan), migrated cells were counted in five random fields.

### 2.13 Western blotting

The lysis buffer containing radioimmunoprecipitation (RIPA) and 1 mM phenylmethanesulfonyl fluoride (PMSF, Beyotime Biotechnology, China) were used for the extraction of protein from HEC-1 cells and Ishikawa cells treated with various concentrations of quercetin. After centrifugation with 12000 g for 20 min at 4°C, the total protein in each group was measured with BCA Protein Assay Kit (Beyotime Biotechnology, China). Then proteins were fractionated in SDS-polyacrylamide gels and moved to blotting membrane (polyvinylidene fluoride, PVDF). Then, the membrane was incubated with the primary antibody at 4°C overnight, followed by 1 h at room temperature with the second antibody. Rabbit anti-human *ISG15* (1:1000, Proteintech, China) and rabbit anti-human *GAPDH* (1:2000, Affinity Biosciences, China) were used as primary antibodies. The second goat anti-rabbit and conjugated with peroxidase were purchased from Biosharp (1:3000, China). Lastly, ECL reagent was applied to visual the protein bands.

### 2.14 Statistical methods

The data were analyzed using SPSS 24.0 and R 3.6.3. The experiments were conducted at least three times, and the results were expressed as mean ± standard deviation. The Student’s t test was used in two groups, and one-way ANOVA was applied to determine the differences in multiple groups. *P* < 0.05 represented statistical significance.

## 3 Results

### 3.1 Identification of UCEC DEGs and CRGs

**Figure 2:**
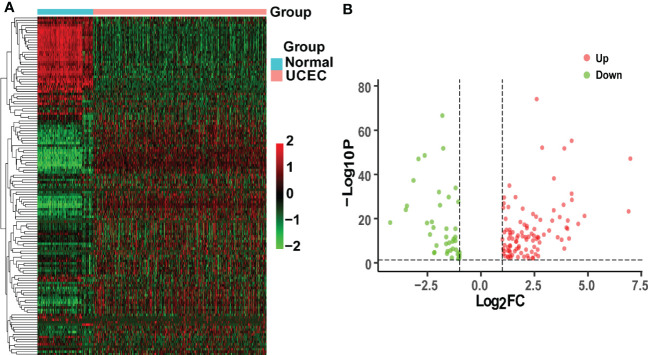
Analysis of intersecting genes in UCEC/COVID-19. **(A)** The heatmap of differential expression about intersecting genes in UCEC/COVID-19. **(B)** The volcano plot depicting 138 DGEs. Red dots and green dots represent differentially expressed up-regulated and down-regulated genes, respectively.

TCGA-UCEC and GTEx cohorts were used to identify DEGs between normal uterus tissues and UCEC samples. A total of 6067 up-regulated genes and 2881 down-regulated genes were obtained by the criteria of |log_2_(FC)| > 1 and *P*< 0.05. Then, we obtained 492 CRGs. Finally, 138 overlapping genes were discovered in the UCEC DEGs and CRGs. Besides, 98 DEGs were up-regulated, while the other 40 DEGs were down-regulated in UCEC (**Figure 2**).

### 3.2 Recognition of quercetin’s targets

**Figure 3:**
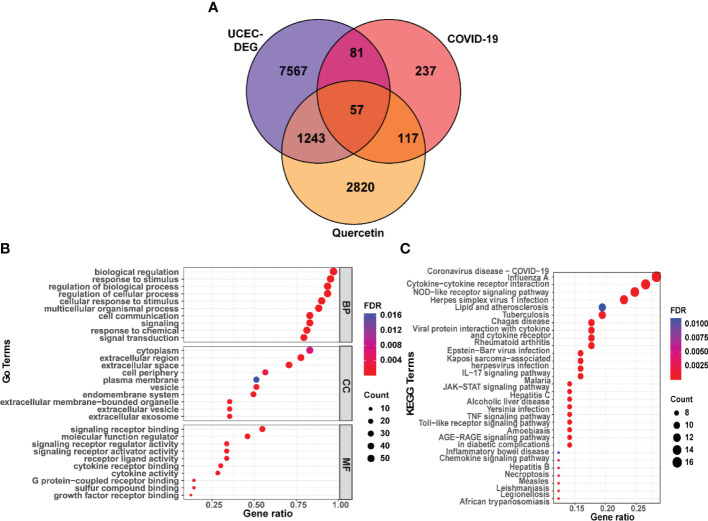
Functional characterization of quercetin against UCEC/COVID-19 intersecting genes. **(A)** Venn diagram describing intersecting genes of quercetin and UCEC/COVID-19. **(B)** GO analysis of intersecting genes of quercetin and UCEC/COVID-19. **(C)** KEGG pathway analysis of intersecting genes of quercetin and UCEC/COVID-19.

A total of 4237 drug targets for quercetin were predicted from KEGG, CTD, SwissTargetPrediction, DrugBank, and TargetNet. Then, the UCEC-DEGs/CRGs and quercetin target genes were analyzed, and 57 core target genes were acquired (**Figure 3A**). Moreover, 43 genes in core targets were up-regulated and 14 genes were down-regulated.

### 3.3 Functional enrichment analysis of core targets

Subsequently, functional enrichment analyses were performed by g:Profiler online tool. The results of GO found that the top BP terms were ‘biological regulation’, ‘response to stimulus’, and ‘regulation of cellular process’ (**Supplementary Table 2**). Besides, GO analysis identified the top CC terms, including ‘cytoplasm’, ‘extracellular region’, and ‘extracellular space’. Moreover, the top MF terms were ‘signaling receptor binding’ and ‘molecular function regulator’ (**Figure 3B**). In addition, the KEGG analysis results showed a significant relationship between 57 genes and ‘coronavirus disease-COVID-19’. (**Figure 3C**).

### 3.4 Construction of target network for quercetin’s core genes

**Figure 4:**
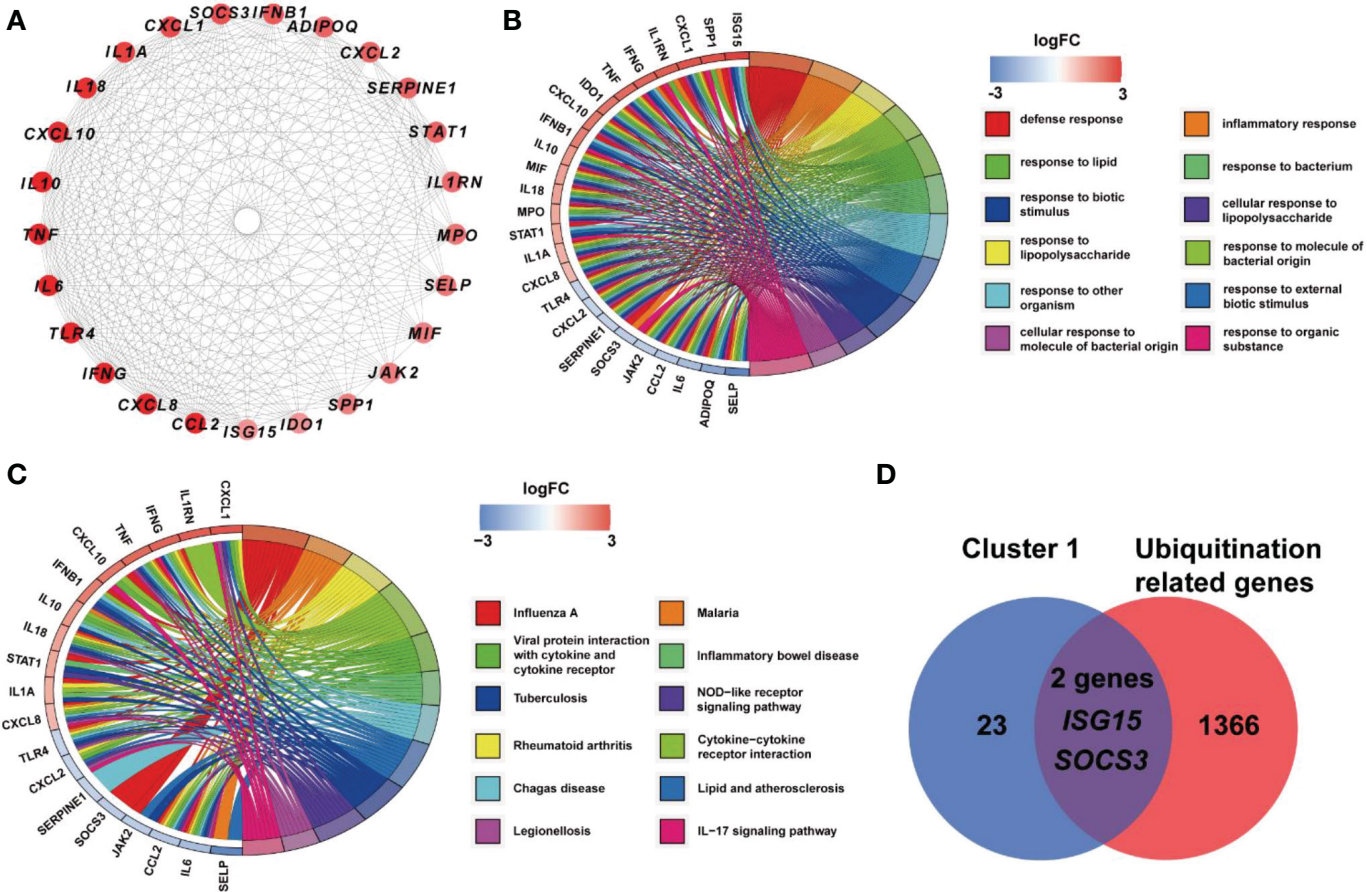
Analysis of cluster 1 from PPI network. **(A)** Cluster 1 contained 25 genes. **(B)** BP results from GO enrichment analysis of Cluster 1. **(C)** KEGG pathway analysis of genes in Cluster 1. **(D)** Two genes in Cluster 1 were URGs.

**Figure 5:**
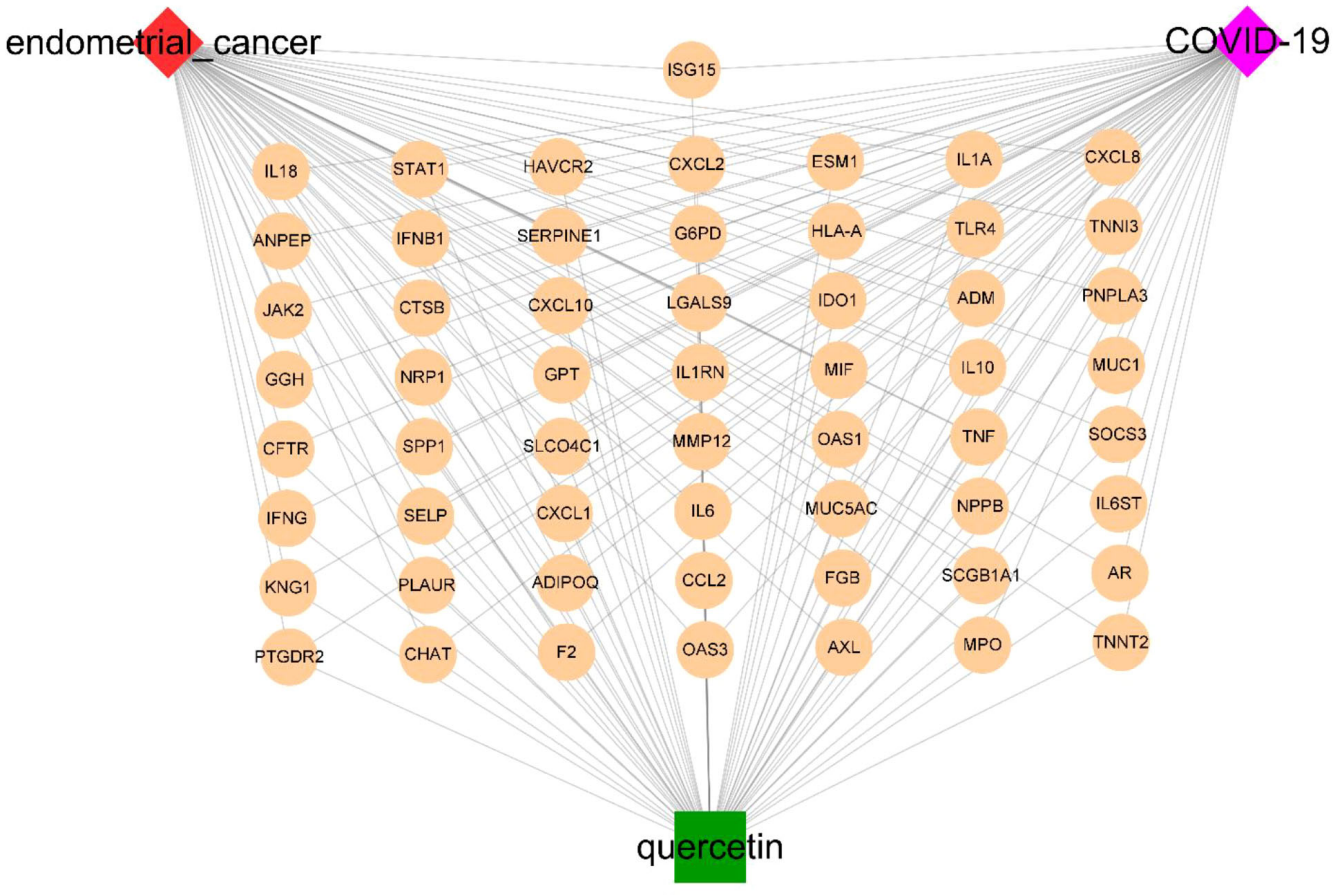
The diseases-drug-targets network of quercetin treatment for UCEC/COVID-19 patients.

Based on STRING platform, PPI network, which contained 57 nodes and 431 edges, was established to explore the mechanism of quercetin for treating UCEC/COVID-19 (**Supplementary Figure 1**). The cluster analysis of the PPI network was performed through MCODE function in Cytoscape 3.6.1 software and two clusters were found (**Table 1**). As shown in **Figure 4A**, cluster 1 consisted of 25 nodes and 236 edges. Then, GO analysis demonstrated cluster 1 was significantly enriched in ‘defense response’, ‘inflammatory response’, and ‘response to biotic stimulus’ (**Supplementary Table 3** and **Figure 4B**). The KEGG pathway analysis showed cluster 1 was significantly enriched in ‘NOD-like receptor signaling pathway’, ‘Cytokine-cytokine receptor interaction’, and ‘IL-17 signaling pathway’ (**Figure 4C**). It is worth mentioning that two genes (*ISG15*, *SOCS3*) in cluster 1 were involved in the ubiquitination processes (**Figure 4D**). As shown in **Figure 5**, the diseases-drug-targets network contained 60 nodes and 171 edges.

**Table 1:** MCODE clusters of hub genes in co-expression networks in UCEC.

Cluster	Score	Gene
Cluster1	19.667	*CXCL10, TNF, IL10, IFNB1, IL6, SPP1, JAK2, IDO1, TLR4, SOCS3, ISG15, CXCL8, IL18, SELP, IL1A, IL1RN, STAT1, IFNG, CCL2, MPO, SERPINE1, MIF, ADIPOQ, CXCL1, CXCL2*
Cluster2	3.000	*SCGB1A1, MUS5AC, MUC1*

### 3.5 Clinical prognostic analysis of target genes of quercetin in UCEC DEGs and CRGs

**Figure 6:**
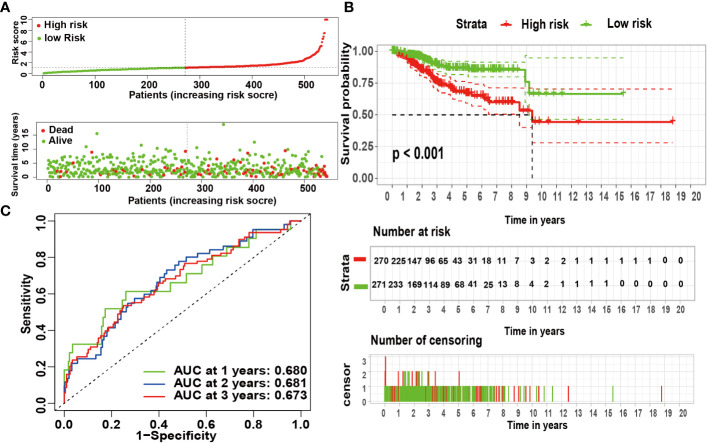
The risk coefficient model of UCEC predicted outcome. **(A)** The distribution of the risk scores of UCEC patients. **(B)** A Kaplan-Meier curve was constructed based on the survival status of the high- and low-risk groups. **(C)** The time-dependent curve showed that the AUC values at year 1, 2, and 3 were separated 0.680, 0.681, and 0.673, respectively.

**Figure 7:**
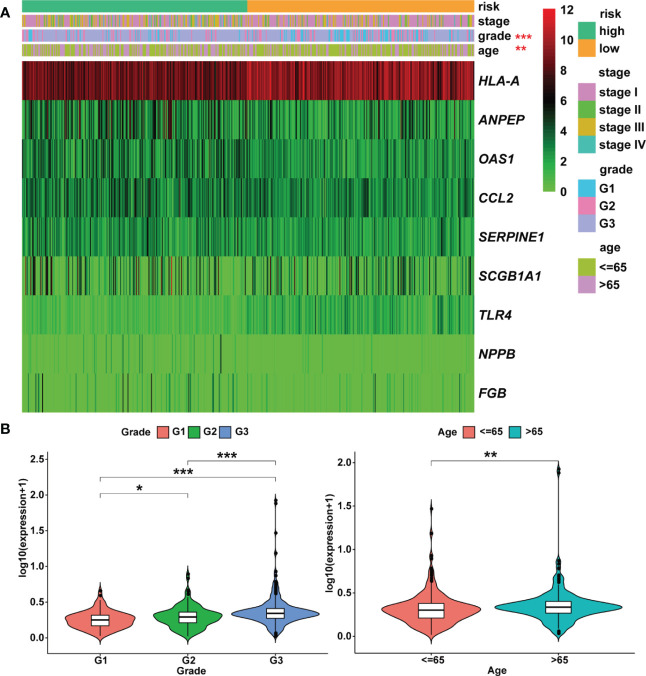
Heatmap and clinicopathologic parameters. **(A)** The clinical heatmap of 9 risk prognostic genes. **(B)** The risk scores were higher in the grade 3 and older patients. * means *P*< 0.05; ** means *P*< 0.01; *** means *P*< 0.001.

According to univariate Cox analysis, 15 genes out of 57 drug targets in UCEC-DEGs/CRGs were significantly associated with patient prognosis (**Table 2**). Subsequently, the multivariate Cox analysis was conducted on the 15 prognostic genes. Nine risk signatures (*ANPEP*,*OAS1*, *SCGB1A1*, *HLA‐A*, *NPPB*, *FGB*, *CCL2*, *TLR4*, and *SERPINE1*) were obtained, and the risk scores of 9 genes were performed (**Table 3**). Next, the UCEC patients were categorized into two distinct groups based on the median risk score (**Figure 6A**). The formula of risk signature was as follow: *ANPEP* expression * 0.00202 + *OAS1* expression * 0.02377 + *SCGB1A1* expression * 0.00129 + *HLA*-*A* expression * (-0.00086) + *NPPB* expression * 0.07108 + *FGB* expression * 0.04111 + *CCL2* expression * 0.01445 + *TLR4* expression * (-0.18252) + *SERPINE1* expression * 0.02068. As shown in **Figure 6B**, OS time was longer in group at low risk. In addition, ROC curves demonstrated the AUC values were 0.680 at 1 year, 0.681 at 2 years, and 0.673 at 3 years (**Figure 6C**). And there were significant differences in grade and age between the high and low risk groups (**Figure 7**).

**Table 2:** Significant genes from univariate Cox regression analysis (*P* < 0.05).

Gene	HR	HR.95L	HR.95H	*P* value
*ANPEP*	1.003	1.001	1.005	0.009
*STAT1*	1.005	1.000	1.009	0.036
*TNF*	1.034	1.014	1.054	0.001
*OAS1*	1.019	1.006	1.033	0.006
*SCGB1A1*	1.001	1.000	1.002	0.006
*SLCO4C1*	1.376	1.077	1.757	0.011
*HLA-A*	0.999	0.999	1.000	0.034
*GPT*	1.022	1.000	1.043	0.047
*NPPB*	1.082	1.025	1.143	0.005
*FGB*	1.033	1.001	1.067	0.045
*CCL2*	1.012	1.006	1.019	0.000
*SOCS3*	1.008	1.003	1.019	0.000
*TLR4*	0.840	0.723	0.977	0.023
*IL6*	1.016	1.004	1.028	0.008
*SERPINE1*	1.019	1.002	1.035	0.024

**Table 3:** Results from multivariable Cox regression analysis.

Gene	Coef	HR	HR.95L	HR.95H	*P* value
*ANPEP*	0.002	1.002	1.000	1.004	0.101
*OAS1*	0.024	1.024	1.009	1.039	0.002
*SCGB1A1*	0.001	1.001	1.000	1.002	0.012
*HLA-A*	-0.001	0.999	0.999	1.000	0.005
*NPPB*	0.071	1.074	1.009	1.142	0.024
*FGB*	0.041	1.042	1.009	1.075	0.011
*CCL2*	0.014	1.015	1.008	1.021	0.000
*TLR4*	-0.183	0.833	0.709	0.979	0.026
*SERPINE1*	0.021	1.021	1.005	1.037	0.010

**Figure 8:**
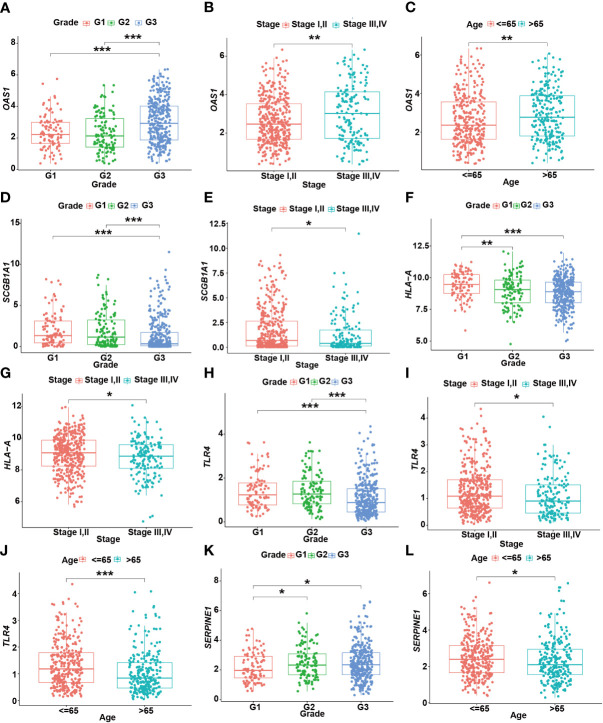
The relationship between prognostic gene levels and clinical characteristics. The relationship between the expression of *OAS1* and grade **(A)**, stage **(B)**, and age **(C)** of UCEC patients. The relationship between the expression of *SCGB1A1* and grade **(D)**, stage **(E)** of UCEC patients. The relationship between the expression of *HLA-A* and grade **(F)**, stage **(G)** of UCEC patients. The relationship between the expression of *TLR4* and grade **(H)**, stage **(I)**, and age **(J)** of UCEC patients. The relationship between the expression of *SERPINE1* and grade **(K)**, age **(L)** of UCEC patients. * means *P*< 0.05; ** means *P*< 0.01; *** means *P*< 0.001.

Moreover, the expression levels of risk signatures of UCEC patients with distinct clinical features were compared. According to the results, *OAS1* was significantly increased in grade 3, stage III/IV, and older age (> 65) groups than that in grade 1/2, stage I/II, and younger (≤ 65) groups, respectively (**Figures 8A–C**). In contrast, the level of *SCGB1A1* was significantly lower in grade 3 and stage III/IV than that in grade 1/2 and stage I/II, respectively (**Figures 8D, E**). Besides, the results showed that *HLA‐A* level was significantly higher in grade 1 and stage I/II than that in grade 2/3 and stage III/IV, respectively (**Figures 8F, G**). In grade 3, stage III/IV, and older age groups, the level of *TLR4* was significantly lower than that in grade 1/2, stage I/II, and younger groups, respectively (**Figures 8H–J**). In addition, the *SERPINE1* was lowly expressed in grade 1 and older age groups than that in grade 2/3 and younger age groups, respectively (**Figures 8K, L**).

### 3.6 Mutation profiles of gene signatures in risk model

**Figure 9:**
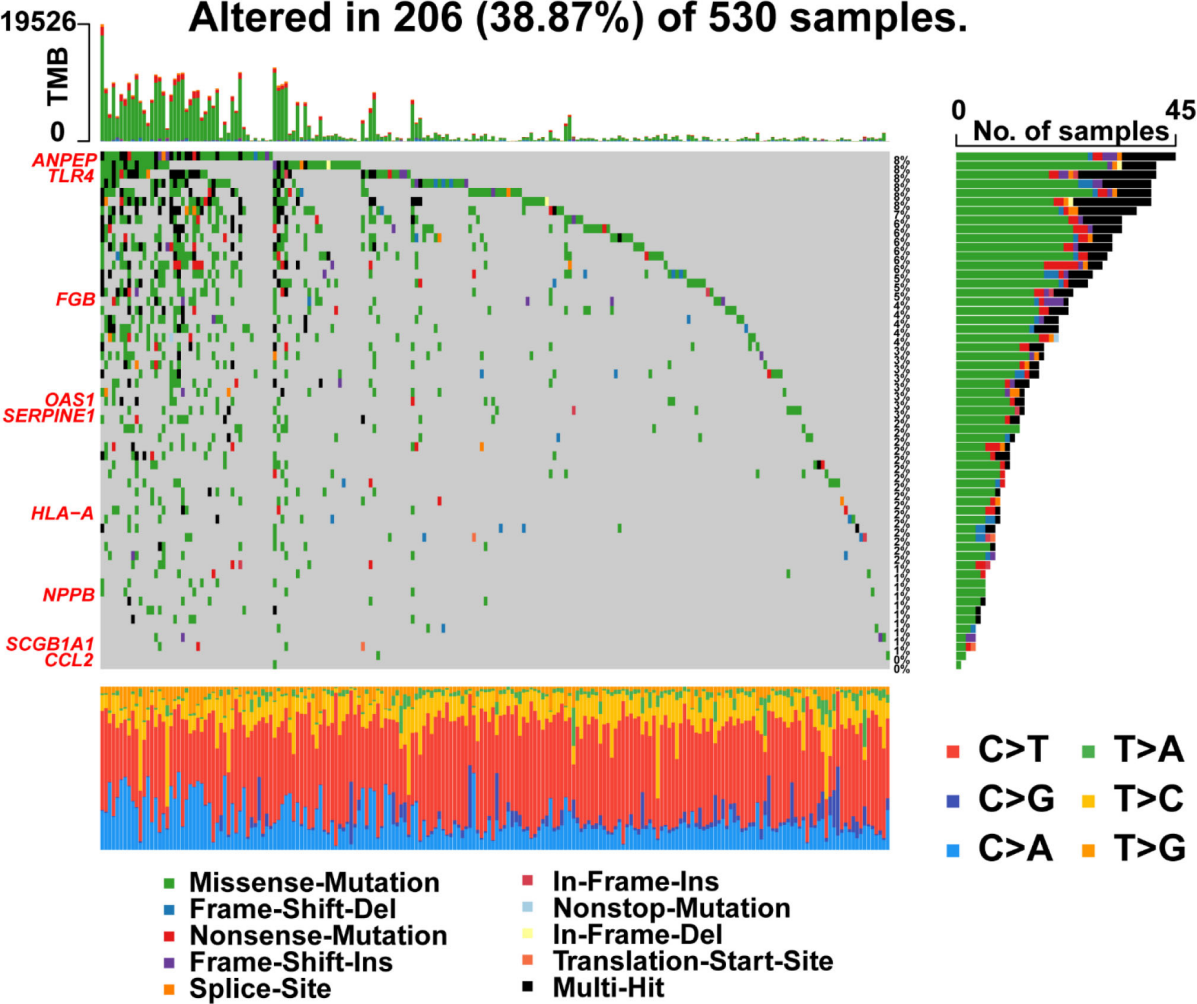
Landscape of mutations in 57 genes in UCEC. The left panel represents 9 prognostic genes which ordered by their mutation frequencies. The bottom panel represents 10 types of mutation.

Furthermore, R package ‘maftools’ was used to analyze and visualize the mutation frequencies of 9 risk signatures. As shown in **Figure 9**, *ANPEP* (8%), *TLR4* (8%) and *FGB* (5%) were found commonly mutated. Mutations in genes including *OAS1* (3%), *SERPINE1* (3%), *HLA*-*A* (2%), *NPPB* (1%), and *SCGB1A1* (1%) were also observed.

### 3.7 Immune landscape of gene signatures in risk model

**Figure 10:**
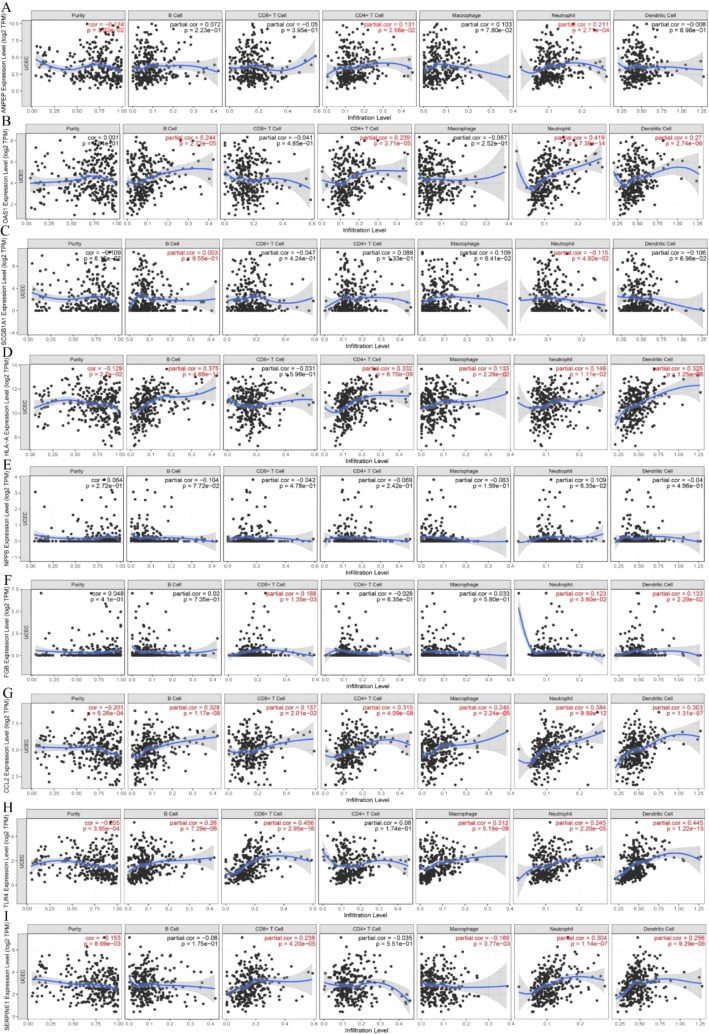
Correlation of 9 prognostic genes with immune cells in UCEC. **(A)** *ANPEP*, **(B)** *OAS1*, **(C)** *SCGB1A1*, **(D)** *HLA‐A*, **(E)** *NPPB*, **(F)** *FGB*, **(G)** *CCL2*, **(H)** *TLR4*, and **(I)** *SERPINE1*.

**Figure 11:**
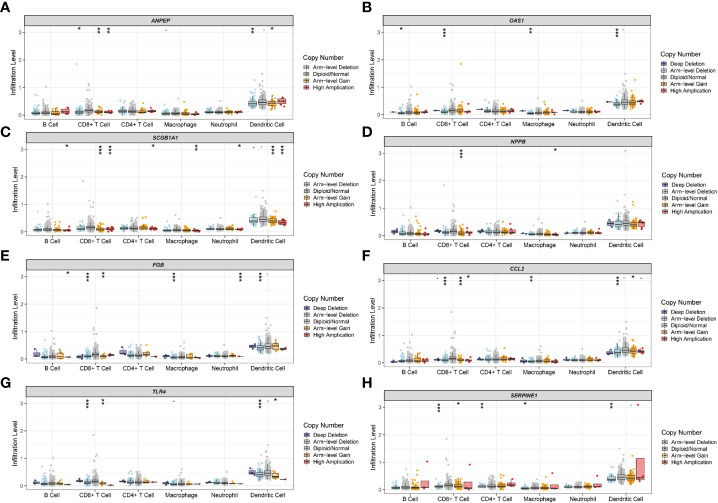
Immune cell infiltration influenced by copy number variation of prognostic genes. **(A)** *ANPEP*, **(B)** *OAS1*, **(C)** *SCGB1A1*, **(D)** *NPPB*, **(E)** *FGB*, **(F)** *CCL2*, **(G)** *TLR4*, and **(H)** *SERPINE1*.

The immune landscape of the risk signatures in UCEC was further investigated *via* TIMER. Based on the results, the level of *ANPEP*, *HLA*‐*A*, *CCL2*, *TLR4* and *SERPINE1* had statistically significant association with tumor purity in UCEC tissues (**Figures 10A–I**). In addition, the correlations were also observed for 5, 4, 4, 4, 8, and 6 risk signatures, corresponding to the B cells, CD8+ T cells, CD4+ T cells, macrophages, neutrophils, and dendritic cells infiltration levels, respectively. Moreover, as shown in **Figure 11**, copy number variation of risk signatures had association with the infiltration of certain immune cells.

### 3.8 The protein levels of gene signatures in risk model

**Figure 12:**
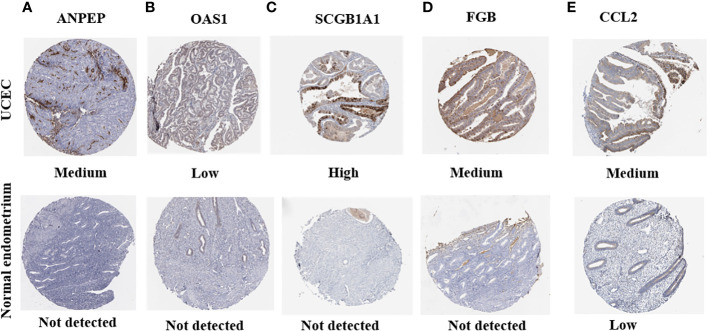
The protein expression of prognostic genes in UCEC tissues and normal endometrial tissues. **(A)** *ANPEP*, **(B)** *OAS1*, **(C)** *SCGB1A1*, **(D)** *FGB*, and **(E)** *CCL2*.

HPA was applied to detect the protein levels of 9 risk signatures in human tissues. Notably, the expression of *ANPEP*, *OAS1*, *SCGB1A1*, and *FGB* could be detected in UCEC tissues. However, the protein levels of these 4 genes were not detected in human normal endometrial tissues. Additionally, medium protein level of *CCL2* was observed in UCEC tissues. But the level of *CCL2* was low in normal endometrial tissues (**Figure 12**).

### 3.9 Molecular docking results

**Figure 13:**
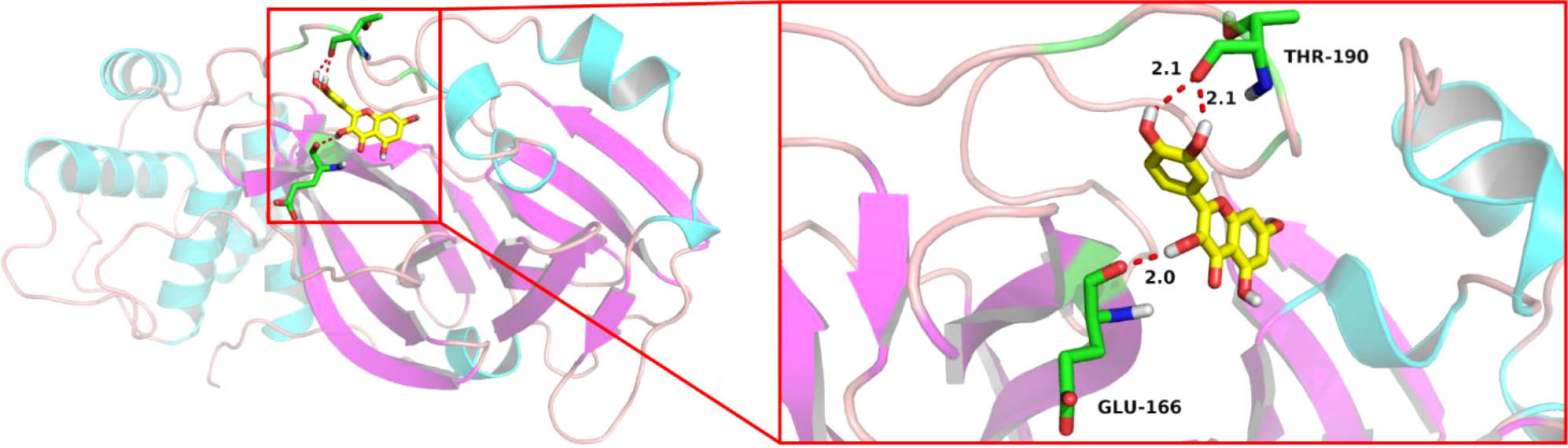
Schematic diagram of molecular docking between quercetin and 7BQY.

Molecular docking was applied to verify whether quercetin docked well with the main protease of SARS-CoV-2. The result showed that three hydrogen bonds formed at acid residues (**Figure 13**). The predicted binding energy of quercetin and main protease was -6.0 kcal/mol, indicating a strong affinity of quercetin and the main protease of SARS-CoV-2. To further explore potential targets in UCEC, molecular docking analyses between quercetin and 9 targets (ANPEP, OAS1, SCGB1A1, HLA‐A, NPPB, FGB, CCL2, TLR4, and SERPINE1) were conducted. As listed in **Table 4**, the molecular docking between quercetin and SERPINE1 had the least binding energy (-6.7 kcal/mol). It was followed by HLA-A (-6.2 kcal/mol) and OSA1 (-6.1 kcal/mol). In addition, docking affinity score between quercetin and ISG15 was -7.2 kcal/mol. And five hydrogen bonds were present in the conformation of quercetin and ISG15 molecular docking (**Supplementary Figure 2**).

**Table 4:** The docking energies and number of hydrogen bonds of 9 targets and quercetin.

Target	Docking score (kcal/mol)	Number of hydrogen bonds
ANPEP	-3.9	1
OAS1	-6.1	3
SCGB1A1	-3.6	2
HLA-A	-6.2	3
NPPB	-2.3	2
FGB	-5.3	3
CCL2	-5.8	3
TLR4	-5.8	2
SERPINE1	-6.7	2

### 3.10 Quercetin inhibited the proliferation and migration of UCEC cells

**Figure 14:**
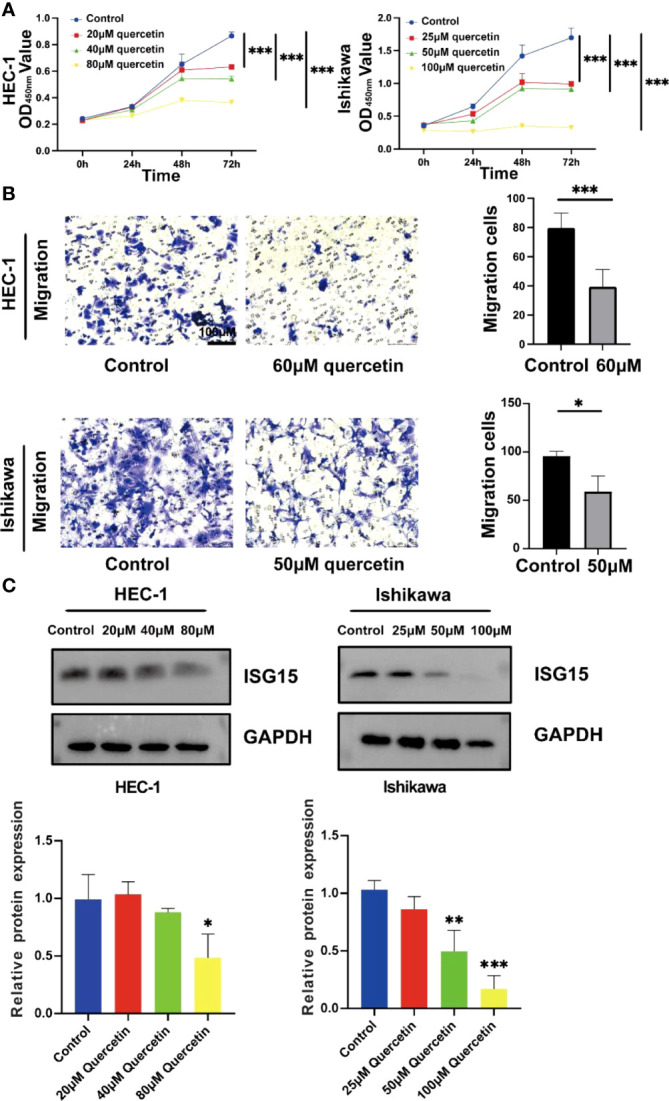
Quercetin inhibits the proliferation and migration of UCEC cells and reduces the expression of *ISG15*. **(A)** Quercetin suppressed viability of HEC-1 and Ishikawa cells by CCK-8 assay. **(B)** Left panel: Transwell assays were used in HEC-1 and Ishikawa cells with or without quercetin. Right panel: Quantitative analyses for Transwell assays. **(C)** Western blotting showed quercetin reduced the expression of *ISG15*. The bar graph showed the ratio of *ISG15* level between quercetin treatment group and control group. * means *P*< 0.05; ** means *P*< 0.01; *** means *P*< 0.001.

The CCK-8 analysis indicated that both HEC-1 and Ishikawa cells were significantly inhibited by quercetin in a dose-dependent manner at concentration from 0 to 80 μM and 0 to 100 μM, respectively (*P*< 0.001, **Figure 14A**). The IC50 values for inhibition of HEC-1 and Ishikawa cells were 67.67 and 48.29 μM at 48 h, respectively. The migration abilities of these two cells were evaluated using transwell migration assay. As shown in **Figure 14B**, compared with controls, the migration of HEC-1 and Ishikawa cells were markedly decreased under quercetin treatment at concentration of 60 and 50 μM, respectively.

### 3.11 Quercetin decreased the expression of ISG15 in UCEC cells

By analyzing TCGA and GTEx cohorts, we found that *ISG15* was significantly elevated in UCEC patients compared to controls (**Supplementary Figure 3**). In order to further investigate the role of *ISG15* in the process of quercetin for treating UCEC patients, the protein level of ISG15 was detected. Our results demonstrated that ISG15 tended to dose-dependently decrease by quercetin treatment in UCEC cells *in vitro* (*P*< 0.05, **Figure 14C**).

## 4 Discussion

During radiation and chemotherapy, many cancer patients might be immunocompromised and their general health might decline (22). It is likely that cancer patients are highly susceptible to COVID-19 during the hospitalization. Quercetin inhibits angiogenesis and affects autophagy in cancer cells to exert its anti-tumor effects (10). Additionally, quercetin could theoretically interfere with SARS-CoV-2 replication (9). Therefore, UCEC patients infected with COVID-19 might benefit from quercetin’s anticancer and antiviral properties. Our study constructed a quercetin-UCEC/COVID-19 network, composed of 57 genes based on UCEC DEGs, CRGs, and quercetin target genes. Functional enrichment analysis showed that quercetin worked mainly by ‘biological regulation’, ‘response to stimulus’, and ‘regulation of cellular process’ against UCEC and COVID-19.

Further, the hub cluster from the quercetin-UCEC/COVID-19 network identified *ISG15*, a gene connected with ubiquitination. The *ISG15*, which was implicated in the pathogenesis of cancer, formed covalent bonds with its target substrates using a cascade of enzymes (E1, E2, and E3) (23). Desai et al. showed that increasing *ISG15* levels in cancer cells exerted pro-tumor effect through ubiquitination-mediated protein regulation (24). Another study found that the elevated level of *ISG15* contributed to immune escape in UCEC, as well as activation of MYC proto-oncogene signaling pathway (15). In the current study, *ISG15* was also elevated in UCEC patients compared to normal controls. Previous study has shown that quercetin inhibited tumor progression by inducing ubiquitination and down-regulating HER2 expression in breast cancer cells (25). Our experiments showed quercetin inhibited UCEC cells proliferation and migration, and quercetin reduced the expression of ISG15 dose-dependently. It is necessary to further explore the role of ubiquitination-related gene *ISG15* in UCEC patients, especially those with COVID-19 infection.

By Cox regression analyses, we identified 9 risk prognostic genes (*ANPEP*, *OAS1*, *SCGB1A1*, *HLA‐A*, *NPPB*, *FGB*, *CCL2*, *TLR4*, and *SERPINE1*) for UCEC. Interestingly, different types of mutations of 9 risk prognostic genes such as nonsense mutation, missense mutation, and multi-hit were also found in UCEC. The high frequency of mutations in *ANPEP*, *TLR4* and *FGB* were observed. However, the specific mechanisms of these mutations on UCEC are not clear now. Our present study revealed that the dysregulation of 9 risk prognostic genes served as an important part in UCEC. A previous study reported that *HLA*-*A* might enhance immune response against tumor mediated by T cells in upper gastrointestinal cancer (26). Allhorn et al. (27) found *TLR4* potentially interacted with hyaluronic acid to activate CD44-mediated signaling, thereby promoting endometrial repair. According to this study, with higher stage and grade of UCEC, the levels of *HLA-A* and *TLR4* were lower, which were significantly associated with longer survival. The results of our study confirm that *HLA-A* and *TLR4* act as tumor suppressor genes during tumorigenesis and tumor progression.

*ANPEP* is a specific ectopeptidase and related to solid tumor growth (28). We confirmed that *ANPEP* associated with poor prognosis and highly expressed in UCEC tissues. A previous study proved that *OAS1* was associated with poor prognosis in pancreatic cancer (29). Our results demonstrated *OAS1* expression in UCEC was positively correlated with stage and grade. The risk of UCEC was also positively associated with *SERPINE1 (* 30). In addition, Wilson et al. (31) found *SERPINE1* could promote endometrial invasion *via* driving *ARID1A* gene mutant. In this study, there was a significant increase in *SERPINE1* expression in the grade 2/3, compared to the grade 1. Thus, we hypothesized that *ANPEP*, *OAS1* and *SERPINE1* contribute to the carcinogenesis of UCEC.

Researchers discovered that *NPPB* played an important role in epithelial ovarian cancer progression (32). Besides, *FGB* was reported to contribute to tumor angiogenesis and metastasis (33). *CCL2* had also been shown to promote tumors and metastases by polarizing macrophages M2 (34). In our study, we also observed that *NPPB*, *FGB*, and *CCL2* were related to poor prognosis in UCEC patients.

It was believed that *SCGB1A1* modulated inflammation and tumorigenesis (35). Furthermore, *SCGB1A1* was considered as a biomarker for ovarian cancers with poor outcomes (36). Similarly to past studies, *SCGB1A1* was considered a risk factor for UCEC prognosis in this work. However, *SCGB1A1* level in grade 3 and stage III/IV was significantly lower than in grade 1/2 and stage I/II of the UCEC, respectively. Further investigation is needed into the complicated mechanism of *SCGB1A1* in UCEC.

Unfortunately, several limitations exist in this study. Additional experiments need to be conducted on cells, animals, and humans to validate the results of this study. Besides, quercetin’s role in mediating proliferation and migration of tumor cells in UCEC patients with COVID-19 through *ISG15*-related pathways needs to be further explored.

**Figure 15:**
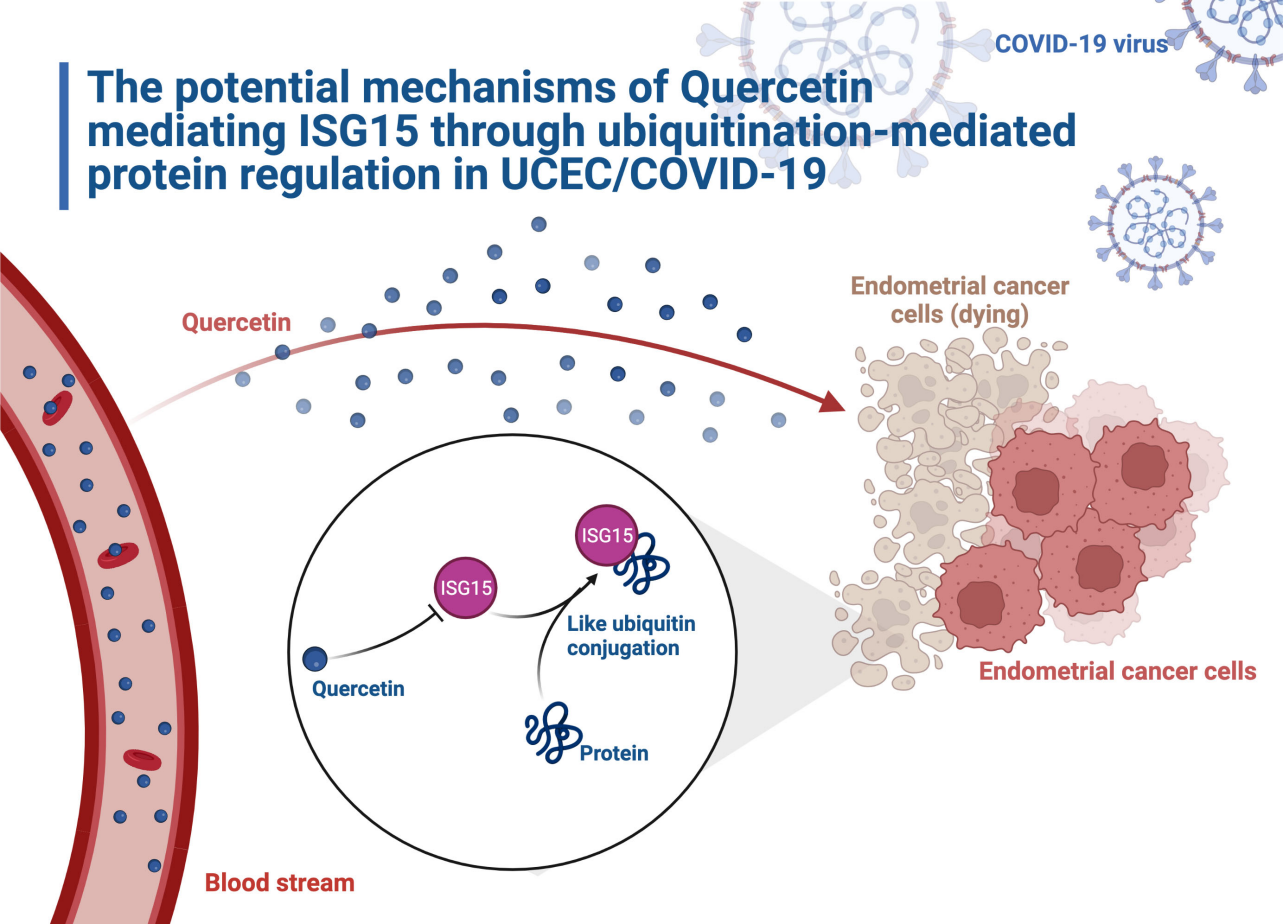
The schematic model illustrates how quercetin plays an anti-cancer role in UCEC.

In conclusion, our findings highlight the feasibility of quercetin for anti-UCEC/COVID-19 by ‘biological regulation’, ‘response to stimulus’, and ‘regulation of cellular process’. Furthermore, network pharmacology identified 57 targets of quercetin that were potentially useful in treating UCEC/COVID-19. The Cox regression analyses suggested that 9 prognostic genes (*ANPEP*, *OAS1*, *SCGB1A1*, *HLA‐A*, *NPPB*, *FGB*, *CCL2*, *TLR4*, and *SERPINE1*) might act critical roles in the treatment of UCEC/COVID-19. Additionally, quercetin could retard the cells proliferation and migration in UCEC and reduce ubiquitination related gene *ISG15* expression (**Figure 15**). This study enriched treatment options for UCEC patients with COVID-19 and provided rationale for strategies for further study of quercetin treatment in UCEC patients infected with COVID-19.

## Data availability statement

The original contributions presented in the study are included in the article/**Supplementary Material**. Further inquiries can be directed to the corresponding author.

## Author contributions

Conceptualization: KL, XZ, and HL. Data analysis and performing experiments: KL, YL, LG, and XX. Investigation and writing: KL and HL. Revising manuscript: YL, LG, and XX. Data supervision and editing manuscript: XZ. All authors contributed to the article and approved the submitted version.
