# Supplementary material for: Discovery of therapeutic targets of quercetin for endometrial carcinoma patients infected with COVID-19 through network pharmacology
Source: Front Oncol. 2023 Mar 8;13:1151434. doi: 10.3389/fonc.2023.1151434 (PMC10031047; doi:10.3389/fonc.2023.1151434)
Supplement: Supplementary file 1 [file DataSheet_1.pdf]

Supplementary Material

Discovery of therapeutic targets of quercetin for endometrial carcinoma patients infected with COVID-19 through network pharmacology

Kehan Li[1†], Hejing Liu[1†], Yibin Lin[1], Liang Gu[2], Xinli Xiang[1], Xueqiong Zhu[1,2*]

*** Correspondence: Xueqiong Zhu,** wzzxq@wzhealth.com

Supplementary Figures

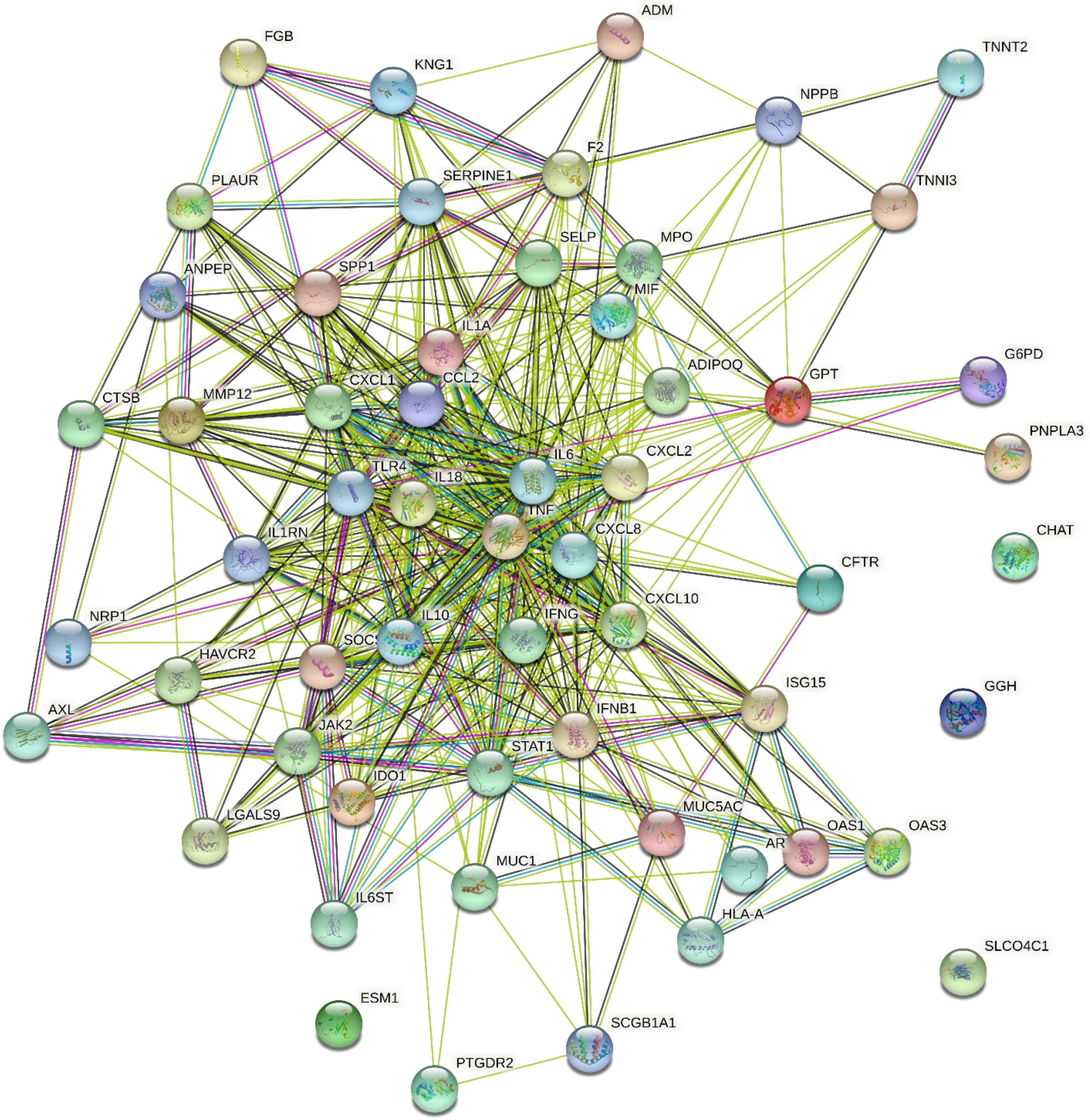

Supplementary Figure 1: PPI network of 57 quercetin targets on UCEC patients infected with COVID-19.

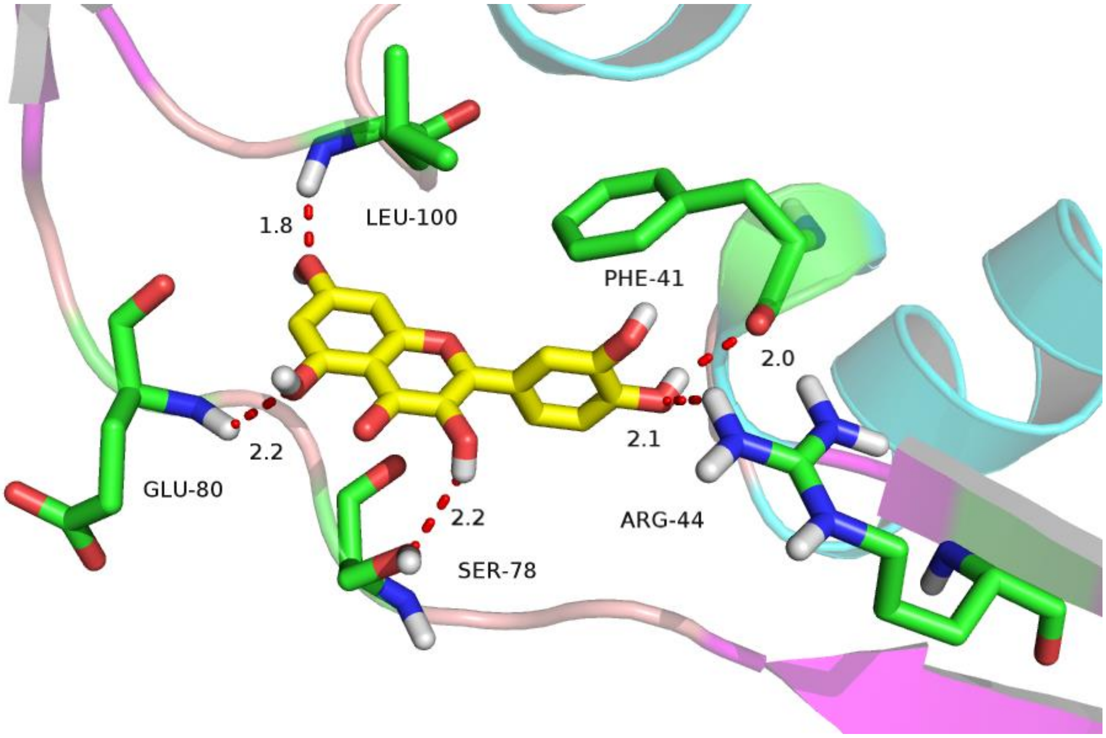

Supplementary Figure 2: Diagram of molecule docking between quercetin and ISG15.

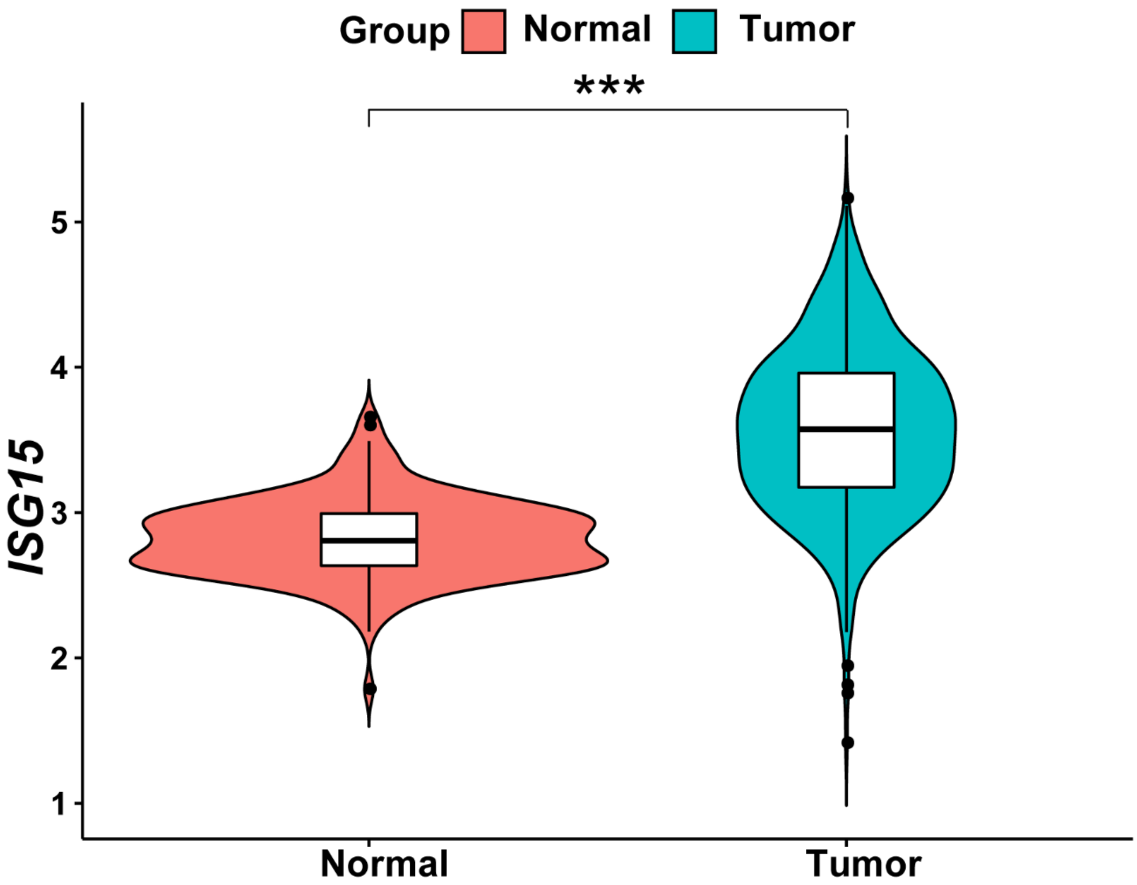

Supplementary Figure 3: *ISG15* is highly expressed in UCEC tissues. *** means $P < 0.001$.
